# Population-Specific Exploration of *MIR146A* Gene Polymorphism in Acute Renal Rejection: A Cross-Sectional, Case–Control Study

**DOI:** 10.3390/ijms27115105

**Published:** 2026-06-04

**Authors:** Nor Elhouda Nacer, Soumia Missoum, Houssem Eddine Ouarhlent, Seddam Hares, Asma Ribouh, Ghania Belaaloui

**Affiliations:** 1 Laboratory of Acquired and Constitutional Genetic Diseases (MAGECA), Faculty of Medicine, University of Batna 2, Batna 05000, Algeria; houdanacer08@yahoo.fr (N.E.N.);; 2 Faculty of Nature and Life Sciences, University of Batna 2, Batna 05078, Algeria; 3 Faculty of Medicine, University of Batna 2, Batna 05000, Algeria; 4 Nephrology Department, University Hospital Benflis Touhami, Batna 05000, Algeria; 5 Biotechnology Research Center (CRBt), Constantine 25000, Algeria

**Keywords:** acute renal rejection, *MIR146A*, rs2910164, HLA-A* mismatch, post-transplant infections

## Abstract

Acute renal allograft rejection (AR) is immune-mediated. Recent evidence highlights some microRNAs as immune modulators. Few and conflicting studies have studied the impact of the *MIR146A* gene single-nucleotide polymorphism rs2910164 (C>G) on AR. We explored the association of rs2910164 with AR in a single-center cohort of 533 kidney transplant recipients (KTRs) by genotyping cases with biopsy-proven late AR (AR group, n = 35) and matched control KTRs without AR (non-AR group, n = 60). Genotyping was performed with real-time PCR. Multivariable logistic regression and Firth penalized logistic regression, as a sensitivity analysis, were used to adjust for confounding factors. Donor–recipient age difference was significantly higher in the AR group than in the non-AR group (23.37 ± 11.29 vs. 14.83 ± 10.54, *p* < 0.001). Recipient mean age in the AR group is lower than in the non-AR group (27.51 ± 10.34 vs. 32.83 ± 9.76 years, *p* = 0.014), while the opposite is observed with the donor mean age (49.57 ± 10.37 vs. 43.27 ± 10.27 years, *p* = 0.005). AR was associated with preformed donor-specific antibodies (DSAs) (45.7% vs. 8.3%, *p* = 0.000, OR = 9.263, 95% CI (2.988–28.720)), and with two HLA-A* mismatches (17.1% vs. 3.3%, *p* = 0.048, OR = 6.000, 95% CI (1.139–31.595)). Moreover, post-transplant viral infections, particularly with CMV and SARS-CoV-2, were associated with AR (*p* < 0.05). However, rs2910164 was not associated with AR across all the tested genetic models (*p* > 0.05). Our study provides population-specific negative association data on rs2910164 and AR. Larger multicentric studies and future meta-analyses are needed to clarify whether any effect is modest or context-dependent.

## 1. Introduction

Kidney allograft transplantation is the optimal treatment for end-stage renal diseases. Acute renal rejection (AR) remains a cardinal immunological cause of long-term kidney transplant failure, which leads to serious concerns in public health. It is a complex, multifactorial process primarily driven by alloimmune responses, involving both cellular and antibody-mediated mechanisms [1]. Understanding AR risk factors would potentially help to estimate its probability and severity, and to guide appropriate selection of immunosuppressive regimens [2]. Various risk factors are associated with AR pathogenesis, including older donor age, cold ischemia time, ABO incompatibility, DSAs, HLA mismatching [3], and viral infections [4].

Recent evidence has also highlighted the role of genetic susceptibility and epigenetic dysregulation [5,6]. For instance, in the analysis of the 2003 UNOS (United Network for Organ Sharing) Registry, graft survival data revealed that HLA mismatches accounted for only 18% of graft loss, while non-HLA immunological factors contributed to 38% of failures even in cases with identical HLAs [7]. This statement proves that other associated non-HLA genetic loci influence long-term graft survival [8]. Many studies have shown an association between AR and single-nucleotide polymorphisms (SNPs) in non-HLA genes that can influence gene expression regulation, cell signaling and proliferation and inflammatory processes related to ischemia–reperfusion injury [9]. Some of them are involved in fibrosis and drug metabolism, reflecting complex genetic interactions and pathways [10].

Emerging evidence underscores the relevance of regulatory non-coding RNAs in modulating alloreactive immune responses. Among these, microRNAs (miRNAs) negatively regulate gene expression by binding the 3′UTR of a target messenger RNA (mRNA), blocking its translation or promoting its degradation [11,12]. They are fundamental regulators of virtually all cellular processes [13] and biological processes such as immune function [14]. Many studies have suggested that miRNAs may serve as significant diagnostic biomarkers and play a potential role in the pathogenesis of AR [15]. For instance, some polymorphisms in miR-196a, *MIR146A* and *MIR499A* genes have been linked to disorders in the immune responses mediated by T cells (TCMR: T-cell-mediated rejection) or B cells (ABMR: antibody-mediated rejection), leading to a twofold increase in the risk of AR [16,17]. In addition, the over-expression of miRNA was noticed in patients suffering from kidney diseases and those experiencing membranoproliferative glomerulonephritis or focal segmental glomerulosclerosis [18].

MiR-146a is emerging as one of the most widely examined miRNAs. It belongs to the miR-146 family [19,20] and participates in the regulation of anti-bacterial immune response [21] and of immune tolerance through regulatory T cells [22], and acts as a key negative feedback regulator of innate and adaptive immune cells. It modulates inflammatory signaling pathways by inhibiting two essential adapter molecules in the TLR pathway, IRAK1 (Interleukin-1 Receptor-Associated Kinase1) and TRAF6 (TNF Receptor-Associated factor 6), which leads to a negative feedback loop to prevent excessive inflammation under normal conditions [19,23]. Like other miRNAs, miR-146a is transcribed as a primary miRNA (pri-miRNA) containing a stem-loop (hairpin) structure. This is cleaved to generate precursor miRNA (pre-miRNA), which is exported to the cytoplasm and processed into a short duplex containing the mature 5p and 3p strands, which are then loaded onto Argonaute proteins to form the effector RNA-induced silencing complex (RISC) that guides target mRNA regulation [24].

The SNP rs2910164 (C>G) is located in the precursor sequence of miR-146a, precisely in the middle of its hairpin stem. The variant G gives a stable G:U pair in the hairpin stem which is necessary for the processing of miR-146a, whereas the variant C gives an unstable C:U mismatch that disrupts processing and reduces the amount of mature miR-146a, diminishing its regulatory effect on its target genes. Hence, the reduced levels of mature miR-146a associated with the rs2910164 C allele lead to reduced suppression of IRAK1 and TRAF6. This results in sustained activation of the NF-κB transcription factor, which in turn promotes the prolonged production of pro-inflammatory cytokines [20,25,26].

Despite significant advancements in genetic research on end-stage renal diseases and AR in association with gene polymorphisms of microRNAs, limited studies in the literature have evaluated the functional consequences of rs2910164 in the progression to AR. Moreover, these studies, conducted in American or Asian populations, showed some contradictions regarding the AR-associated genotypes [18,27,28] or used a “proxy SNP” to show the association [17]. Therefore, the goal of the present study was to further investigate the association of *MIR146A* gene polymorphism with AR occurrence in a North African population. Since AR is a multifactorial process involving both immunologic and non-immunologic determinants, we adopted an integrative approach to evaluate not only the association between rs2910164 and AR susceptibility, but also the potential contribution of additional clinical and biological cofactors within this complex condition.

## 2. Results

### 2.1. Demographic Profiles and Clinical Features

Table 1 summarizes the main clinical and demographic parameters of our cohort. All rejection events included in the analysis were late-AR episodes (≥6 months post-transplant). In the AR group we had 25 ABMRs and 10 TCMRs. Our results showed that despite efforts to match the groups based on age, sex, and residence, a significant difference in age was still observed between KTRs in the AR group and in the non-AR group (27.51 vs. 32.83 yr, respectively, *p* = 0.014) (Table 1). This residual difference in age is due to the fact that age matching was performed by category rather than by exact age. It indicates that age remained a potential confounding variable, which was therefore taken into account in the logistic regression analysis.

Donor mean age and donor–recipient age difference were higher in the AR group than in the non-AR group (49.57 vs. 43.27 yr and 23.37 vs. 14.83, *p* = 0.005 and *p* < 0.001, respectively). Similarly, recipient–donor couples where recipients are older than donors are more associated with AR (OR = 5.333, 95% CI (1.454–19.559), *p* = 0.007) (Table 1).

We did not find any significant difference between the AR group and the non-AR group regarding percentages of both genders, Donor-recipient sex match or in Donor-recipient sex match combinations. However, we noticed that donors are more frequently females in both groups (approximately 60% of cases) whereas recipients are more frequently males (60% of cases) (Table S1).

We did not find any significant association between the occurrence of AR and the remaining factors (*p* > 0.05): recipient Body Mass Index (BMI) and donor–recipient relationship (Table 1), the presence of pretransplant medical disorders, pretransplant blood transfusions and dialysis duration before transplantation (Table S1).

On the other hand, the percentage of couples who have two mismatches (2 MM) in HLA-A* was significantly higher in the AR group than the non-AR group, with a six-times increase in the risk of having an AR (17.1% vs. 3.3%, OR = 6 (1.139–31.595), *p* = 0.048). The same observation was made for the percentage of recipients with preformed DSAs, which was significantly higher in the AR group than in the non-AR group (45.7% vs. 8.3%, OR = 9.263 (2.988–28.720), *p* < 0.001) (Table 1).

Finally, we found that having a post-transplant viral infection is more frequent in the AR group than in the non-AR group, with a 10-fold increase in the risk of having an AR (42.85% vs. 6.67%, OR = 10.5 (3.1–35.6), *p* < 0.001) (Table 1). This risk is especially pronounced with CMV infections (14.3 vs. 0%, OR = 30.3 (1.7–540), *p* = 0.003) and SARS-CoV-2 infection (22.9 vs. 1.7%, OR = 22.4 (2.6–193.3), *p* = 0.002) (Table S1).

### 2.2. Allelic Frequencies and Genotype Distribution

The G allele frequency was higher in the AR group (70.0%) compared to the non-AR group (64.1%), while the C allele was more prevalent in the non-AR group (35.9%) than in the AR group (30.0%). However, none of these differences reached statistical significance (*p* > 0.05). We found that rs2910164 was in HWE in both cases (AR) and controls (non-AR), as shown in Table 2. Additionally, genotypes’ frequencies within the AR group were 51.4%, 37.1% and 11.4% for the genotypes GG, CG and CC, respectively, while in the non-AR group, the CG genotype was more frequent (48.3%), followed by GG (40%) and CC (11.7%) (Table 3).

### 2.3. Acute Renal Rejection Risk and rs2910164 Polymorphism in the Whole Cohort

The distribution of rs2910164 genotypes in cases and controls and their association with AR according to different genetic models are represented in Table 3. The univariate analysis showed no significant association between rs2910164 and AR across the codominant, dominant, recessive, and overdominant genetic models. To account for the potential influence of covariates previously found to be associated with AR (donor–recipient age difference, post-transplant viral infections, HLA mismatches and DSAs; Table 1), we then performed a multivariate logistic regression analysis. This was possible because multicollinearity diagnostics showed very low variance inflation factors (VIF range: 1.062–1.142) and low condition index values (maximum: 5.024), indicating the absence of problematic multicollinearity among the predictors included in the adjusted model. After adjustment for these factors, no genetic model showed a significant association with AR. This finding was consistent when using either standard logistic regression or Firth penalized logistic regression as a sensitivity analysis, indicating that rs2910164 was not independently associated with AR in our cohort (Table 3). To complement these findings, an approximate sensitivity power analysis was performed using QUANTO under the codominant model. The estimated power was 61.1% for an odds ratio of 2.0, and 83.6% for an odds ratio of 2.5, indicating that the study had adequate power only to detect relatively large effects.

### 2.4. Acute Renal Rejection Risk in the Stratified Population

To further explore factors influencing AR, we stratified our AR group into “ABMR” AR (n = 25) and “TCMR” AR (n = 10) subgroups. Comparison of each AR subgroup with the non-AR group (n = 60) revealed the persistence of the differences previously detected before stratification: participants’ ages, preformed DSAs and post-transplant viral infections (Table 4). However, these differences seemed to be observed mostly in the “ABMR” AR subgroup: KTR mean age in the “ABMR” AR group was significantly lower than in the non-AR group (26.92 vs. 32.83 yr, respectively, *p* = 0.016). Donor mean age and donor–recipient age difference were higher in the “ABMR” AR subgroup than in the non-AR group (49.57 vs. 43.27 yr and 23.37 vs. 14.83, with *p* = 0.005 and *p* < 0.001, respectively). Likewise, recipient–donor couples where recipients are older than donors were more associated with “ABMR” AR (OR = 5.75, 95% CI (1.231–26.858), *p* = 0.009). Regarding post-transplant viral infections, they were more frequent in the AR subgroups than in the non-AR group, but the difference reached significance only for the “ABMR” AR, with a 13-fold increase in the risk of having an AR (48% vs. 6.67%, OR = 12.923 (3.584–46.592), *p* < 0.001). However, preformed DSA in KTRs was significantly higher in both the “ABMR” AR and “TCMR” AR subgroups compared to the non-AR group (40% and 60% vs. 8.3%, with OR = 7.333 (2.174–24.737), OR = 16.5 (3.462–78.650), respectively, *p* = 0.001). Finally, we did not find any significant differences between AR subtypes and the non-AR group across the other demographic, clinical, or genetic variables.

## 3. Discussion

In this study, we explored the association of *MIR146A* gene polymorphism rs2910164 with AR risk, alongside other potential confounding risk factors, using a multivariate analysis. Our results did not show any significant association of rs2910164 with AR (Table 3) even after adjustment for the potential confounding factors that were found in the univariate analysis: donor–recipient age difference, preformed DSAs and HLA mismatches, and post-transplant viral infections (Table 1). The absence of genetic association persisted also after stratification by AR subtype (“ABMR” or “TCMR”). To the best of our knowledge, this genetic study is the first to be conducted in a North African population.

In our cohort, the absence of association between rs2910164 and AR was observed under all the genetic models (codominant, dominant, recessive, and overdominant) after adjustment in conventional multivariable logistic regression. This finding remained unchanged when the analysis was repeated using Firth penalized logistic regression. Because this method is less sensitive to small-sample bias and sparse-data issues, the consistency of these results strengthens the interpretation that no independent association could be demonstrated in this cohort after adjustment for the available confounding factors. However, given the sample size of this monocentric study, more modest associations cannot be completely ruled out. Accordingly, our findings should be interpreted as arguing against a major effect of this variant rather than proving the absence of any effect. This perspective is important when comparing our results with previous studies, which have reported inconsistent findings and differed substantially in population characteristics, allele distribution, rejection timing, and study design.

Because no functional analysis of miR-146a expression or related immune pathways was performed, the present work should be interpreted strictly as a genetic association study, and a deeper interpretation of the biological significance of the negative association cannot be made from these data alone. Therefore, the absence of a significant association in our cohort should not be interpreted as evidence that rs2910164 has no biological effect. At the same time, the fact that several established clinical cofactors behaved as expected in our cohort suggests that the dataset captured clinically meaningful determinants of AR, thereby supporting the interpretation that rs2910164 did not emerge as a major independent determinant of AR in this setting. In addition, given the specific clinical and phenotypic characteristics of our cohort, any true effect may still be modest and context-dependent, and could therefore have been obscured by additional sources of heterogeneity, as suggested by the variability observed across the previous studies discussed below.

To date, a few genotype studies have explored rs2910164 association with AR using different methodologies, and showed heterogeneous findings. Abbasi et al. [27] reported results from an Iranian cohort of 100 KTRs (34 AR and 66 non-AR) showing that the CC genotype is significantly enriched in the AR group compared to the non-AR group. At the allele level, they reported that C is a risk allele for AR, while the G allele is protective. Misra et al. [28] conducted an association study in a North Indian cohort of 350 KTRs, but the controls were healthy. They found that the GG genotype is enriched in the AR group. Consistently, their survival analyses showed that the GG genotype has the worst survival. Similarly, Oetting et al. [17] reported the association of the same SNP with AR in an African American (AA) kidney transplant cohort, but not in a European American (EA) cohort. In this study, the authors did not genotype rs2910164 directly; they used rs2961920 as a proxy for rs2910164 as they are in high LD in both populations. Additionally, the authors did not compare genotype frequencies but used time-to-event (AR) Cox models. They found that the proxy rs2961920 A allele is associated with greater AR risk; *p* ≈ 6 × 10^−4^. Using the application LDlink, we found that rs2961920-A is correlated with rs2910164-G with perfect LD (D′ = 1, r^2^ = 1) in the evaluated reference populations AA and EA. This allowed us to infer that rs2910164-G is the one associated with AR in these populations. In accordance with Oetting et al.’s findings, another study conducted by Wysoczańska et al. [18] on a European cohort (108 Polish KTRs and 125 matched healthy controls) did not show any association between rs2910164 and AR. From these available studies, we notice that the presence and the direction of association for rs2910164 diverge by ancestry and by study design. The discrepancies can be explained by the following: (i) There were differences in study design (comparing AR vs. non-AR patients or AR vs. healthy controls) and differences in sample size between studies. (ii) There was phenotype heterogeneity, as some studies (including ours) used biopsy-confirmed AR [17,28], while others did not clearly define AR criteria [18,27]. Additionally, donor types are not the same: cadaveric only [27], living only [28], or both types [17,18]. In addition, our cohort consisted of relatively young recipients compared to those reported in the previous studies [17,18,27], which may also have influenced the observed association, since stronger overall alloimmune responsiveness in younger recipients could mask the modest effect of a single regulatory polymorphism. (iii) Applicability of proxy SNPs across various datasets may differ, because allele correlations depend on ancestry LD. (iv) There were population differences in allele frequencies. If we compare G allele frequency in controls we have: 64.1% in our North African cohort, 38% in North Indians [28], 63.6% in Iranians [27], 80% in Polish controls [18], 76% in EAs and 57% in AAs controls [17]. Such allele frequency variation may influence both statistical power and local haplotype context, potentially modifying detectable effect sizes across cohorts. Additionally, North African populations are genetically admixed; therefore, differences in allele frequencies and local LD patterns may contribute to variability in association signals and to limited portability of proxy-based findings across ancestries. Beyond genetics, differences in epigenetics may influence the functional impact of this SNP on transplant outcomes. (v) Another source of discrepancy across studies is the heterogeneity in the timing of AR. Previous studies assessed rejection over markedly different post-transplant periods, ranging from the first 3 to 6 months [18,27,28] to mixed 12-month or all-time AR analyses [17], whereas our cohort was restricted to late AR. Because early and late rejection may differ in their clinical and immunologic context, comparisons across these studies should be interpreted with caution. (vi) Finally, variations in allele coding may be misleading. Indeed, allele reporting for rs2910164 varies across studies: some of them used major/minor (common/rare) allele or “wild-type/mutant” wording which is population-dependent, while others (like us) used Reference (REF) vs. Alternate (ALT) allele wording which is genome-build-dependent, and is adopted in the dbSNP nomenclature “rs2910164 (C>G)”.

To gain deeper insight into the immunological mechanisms underlying AR, we performed a stratified analysis separating AR cases into TCMR and ABMR subtypes (Table 4). We found a persistent association of both KTRs’ and donors’ ages with AR, but only in the ABMR group. However, in the literature younger KTRs tend to have more AR of both the TCMR [29] and ABMR subtypes [30], and older donors tend to have an increased risk of TCMR [3]. Our results may be explained by the overrepresentation of the ABMR group in our cohort or by the existence of other confounding factors like cold ischemia time or recipient preimmunization. We also found a persistent influence of preformed DSAs in both TCMR and ABMR subtypes, suggesting that DSAs may operate as upstream amplifiers of alloimmunity, inducing possible sequential development of TCMR after ABMR. Additionally, “pure TCMR” groups can still include cases with antibody involvement that is under the detection threshold or not fully captured. Indeed, Banff classification updates explicitly recognize intermediate or “probable” antibody-mediated phenotypes and diagnostic complexity [31]. Alternatively, preformed DSAs can co-exist with a broader sensitization and behave like a risk-enrichment marker, not a purely mechanistic ABMR label [32]. Our results also showed a persistent association of AR with post-transplant viral infections in both TCMR and ABMR subtypes. These infections may promote AR through the activation of inflammatory gene expression, the cross-reactivity of antiviral memory T cells resulting in “heterologous immunity”, or a reduction in immunosuppressive therapy, often undertaken during treatment for viral infection [4].

In our study, several established demographic and clinical cofactors were associated with AR. This supports the internal clinical coherence of the cohort and provides context for interpreting the absence of an independent association with rs2910164. For instance, the association between donor–recipient age difference and AR supports the concept of DRAG, in which an older-donor-to-younger-recipient combination may confer higher rejection risk [3,33]. Additionally, HLA analysis showed that only HLA-A* mismatch was associated with AR. This result may reflect the structure of our dataset, including consanguinity, analysis of only three loci (A, B, and DR), the late-AR profile of the cohort, possible underestimation of HLA-DQ mismatch, and limited variability in DR mismatching [34,35,36,37]. Preformed DSAs and post-transplant viral infections were also associated with AR.

By contrast, sex, BMI, pretransplant comorbidities, donor–recipient biological kinship, dialysis duration before transplantation and prior blood transfusions were not associated with AR in our cohort. For many of these factors, published findings remain inconsistent [38,39,40,41].

The strengths of our work include the evaluation of AR risk in a North African cohort, a population that is underrepresented in transplant genetics. Additionally, we utilized clinically meaningful rejection phenotyping with subtype stratification (TCMR vs. ABMR), we chose a biologically active gene and a functional SNP, and we matched all the cases and controls. Moreover, our analysis integrated key immunologic and post-transplant determinants, such as age, preformed DSAs and viral infections, which improves biological interpretability and clinical relevance, especially with the use of logistic regression analysis in order to address concerns about potential AR risk confounding factors, as well as the use of Firth penalized logistic regression as a sensitivity analysis to account for possible sparse-data bias. The concordance between the two regression approaches supports the robustness of the adjusted negative finding, although the limited sample size still warrants cautious interpretation. Furthermore, standardized genotyping with explicit allele nomenclature and transparent reporting enhances reproducibility and facilitates comparisons with prior studies. However, our study does have some limitations that we attempted to mitigate. Firstly, the sample size was limited because the work was conducted at a single transplantation center, which constrained the number of eligible KTRs and AR events available for analysis. To reduce the impact of sparse-data bias and small-sample instability, we performed Firth penalized logistic regression, which yielded results consistent with standard logistic regression. Nevertheless, the approximate sensitivity power analysis indicated limited power to detect small-to-moderate genetic effects, with adequate power reached only for relatively large effects under the codominant model. Therefore, although no significant association was identified, a type II error cannot be excluded and more modest effects may have remained undetected. Secondly, because of the cross-sectional study design, biopsies were performed on clinical indication, so subclinical rejection cannot be fully excluded, particularly among DSA-positive patients. To partially account for this, we included DSA status in the multivariable adjustment. It should also be noted that previous studies evaluating rs2910164 and AR were subject to a similar limitation [17,28], which should be considered when interpreting discrepancies across studies. Thirdly, our study design does not support firm conclusions regarding possible subtype-specific effects of rs2910164 across ABMR and TCMR, and the negative result should therefore be interpreted in the context of a mixed late-AR cohort. Finally, candidate-gene studies inherently risk overlooking other important genetic variants since they concentrate solely on selected SNPs.

## 4. Patients, Materials and Methods

### 4.1. Patient Recruitment and Sampling

In this cross-sectional study, we started working from a cohort of 533 kidney transplant recipients (KTRs) treated at the Nephrology and Transplantation Medicine Center in Batna University Hospital, Eastern Algeria, during the time period between 2014 and 2021. We found 35 cases with biopsy-proven AR diagnosed on indication biopsy, for whom we selected a control group of 60 KTRs without documented biopsy-proven AR. The decision to perform allograft biopsy was based on the occurrence of unexplained graft dysfunction with an increase in serum creatinine compared with baseline nadir serum creatinine, after excluding alternative causes such as urinary obstruction and infection (Table S1). Cases and controls were age-, sex- and residency-matched. Each AR case was matched to one to two controls without AR according to the following criteria: sex (exact match), residency (exact match), and age category (<18, 18–59, or >60 years).

Hence, the total 95 KTRs (57 men and 38 women) are divided into two groups:

(1) AR group: A total of 35 KTRs with one or more AR episodes during their follow-up;

(2) Non-AR group: A total of 60 KTRs without AR. They were matched to AR KTRs by age, sex and residency, and had stable graft function.

**Figure 1:**
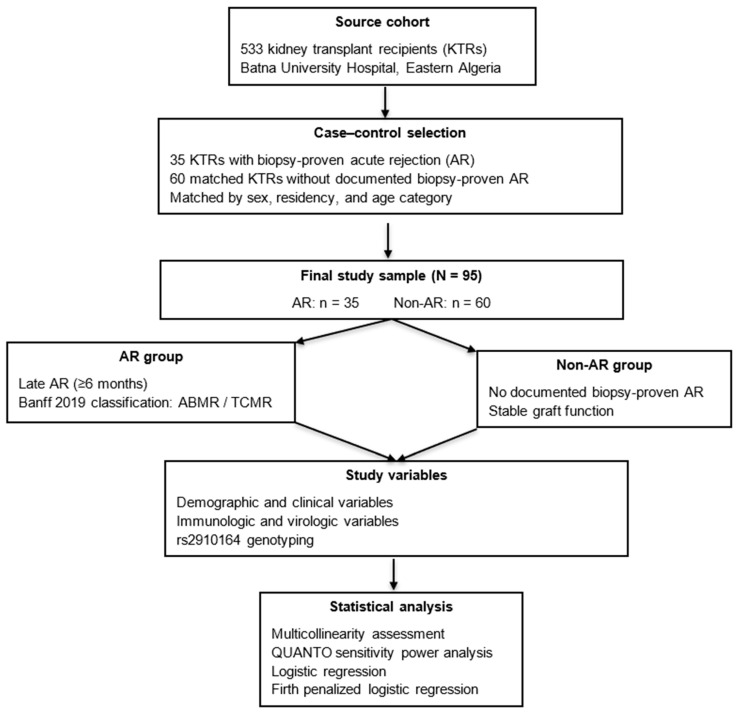
Flow diagram of the study design and analytical workflow.

The non-AR group was defined by the absence of documented biopsy-proven AR on indication biopsy and by stable serum creatinine during follow-up. The study design and main analytical workflow are summarized in Figure 1.

All patients received kidney transplants from living donors (82 related and 13 unrelated) with 0–6 HLA mismatches. The AR group was initially identified clinically by the rise in serum creatinine levels in the absence of other pathologies and was subsequently confirmed histologically based on the Banff classification [42]. Rejection episodes were classified according to this classification as antibody-mediated rejection (ABMR) or T-cell-mediated rejection (TCMR). All patients in this group had late AR, with rejection occurring at least six months after transplantation.

The cohort was classified according to the relationships between donors and recipients to evaluate the influence of genetic similarity on outcomes. Spouses were categorized as unrelated, while first-degree relatives were separated from second- and third-degree relatives. Donor–recipient age imbalance was included to assess the direction of the donor–recipient age gradient (DRAG) by distinguishing transplants in which the donor was older than the recipient from those in which the donor was younger than the recipient [3,33]. Preformed DSAs were assessed using bead-based immunofluorometry with Luminex Single Antigen Bead technology, and positivity was interpreted according to the immunology laboratory’s validated positivity threshold for this assay. Post-transplant viral infections, including BK virus, CMV, and SARS-CoV-2, were identified from the medical records based on documented post-transplant episodes of infection. KTRs’ immunosuppressive therapy comprised corticosteroids and calcineurin inhibitors (tacrolimus/cyclosporine).

Ethical approval was given by the institutional ethical review board of CHU Benbadis (IRB number CE/CHUC/30/03/202), and all procedures were conducted in accordance with the Declaration of Istanbul on Organ Trafficking and Transplant Tourism and the Declaration of Helsinki and its subsequent amendments. We followed the Strengthening the Reporting of Observational Studies in Epidemiology (STROBE) statement guidelines for observational research reporting.

Informed written consent was obtained from all subjects involved in the study. Measures were taken to safeguard participants’ information: data were anonymized to ensure confidentiality, personal identifiers were removed prior to data analysis, and access to the data was restricted.

### 4.2. Genotyping Assay and SNP Selection

Peripheral blood (5 mL) was collected in ethylenediaminetetraacetic acid (EDTA)-treated tubes from AR and non-AR groups and stored at −20 °C until DNA extraction. Genomic DNA was extracted from all blood samples using PureLink^®^ Genomic DNA Mini Kits (InvitrogenTM, part of Thermo Fisher Scientific, Waltham, MA, USA) following the manufacturer’s protocol. The purity and quantity of extracted DNA were evaluated with the Thermo Fisher Scientific Nanodrop 8000.

Selection of the studied SNP (rs2910164) was based on: (i) the replication of its association with AR through independent studies and ethnicities [17,27,28], (ii) its influence on pri-miRNA processing and relevance to inflammation [20,25,26], (iii) its substantial minor-allele frequency (MAF) across various populations (0.23–0.38), providing adequate clarity while minimizing power loss due to rarity [43], (iv) the effect size in previous studies seeming large enough for it to be worth testing, showing non-trivial odds ratios (OR) [27,28], and (v) the fact that it can be robustly genotyped by real-time PCR.

Allelic discrimination of rs2910164 (C>G) was performed using real-time PCR genotyping with a TaqMan^®^ 5′ nuclease assay (Applied Biosystems^TM^ 7500 Real-Time PCR System) according to the manufacturer protocol detailed in (https://assets.fishersci.com/TFS-Assets/LSG/manuals/MAN0009593_TaqManSNP_UG.pdf, accessed on 15 January 2026). PCR conditions included an initial cycle at 60 °C for 30 s followed by one cycle at 95 °C for 10 min and finally 40 cycles at 95 °C for 15 s and at 65 °C for 1 min.

Genotyping quality control was based on the final per-sample genotype call generated by the instrument software. Samples initially classified as undetermined were re-genotyped; this occurred in 8 cases. On repeat analysis, all 8 samples achieved an accepted genotype call with a high software-assigned quality score (mean ≥ 99%) and were retained. This resulted in a final genotyping call rate of 100%.

### 4.3. In Silico Proxy Allele Mapping

In order to interpret proxy-based findings, we assessed linkage disequilibrium (LD) and correlated alleles between rs2961920 and rs2910164 using LDlink (LDpair) [44] based on 1000 Genomes Phase 3 reference populations (European American and African American).

### 4.4. Statistical Analyses

The Chi2 test was used to test our cohort for Hardy–Weinberg equilibrium (HWE). We used the same test to perform a univariate analysis of AR association with categorical variables (sex, HLA mismatch, post-transplant infections, recipient anti-HLA antibodies and genotypes). We used Fisher’s exact test when expected counts were too small (less than 5) after data stratification by age and by sex. Then, we studied the association of AR with genotypes using binary logistic regression to provide adjusted odds ratios (ORs) and 95% confidence intervals (CIs). Covariates included in this multivariable model were selected from variables showing an association with AR in univariate analysis at *p* < 0.05. Accordingly, the final model included donor–recipient age difference, post-transplant viral infections, mismatches, and preformed DSAs in addition to the genotype variable. Multicollinearity among these predictors included in the final adjusted model was assessed using linear regression collinearity diagnostics. Given the relatively limited number of AR events, and to reduce the risk of sparse-data bias, Firth penalized logistic regression was additionally performed as a sensitivity analysis to assess the robustness of the findings. In addition, an approximate sensitivity power analysis was performed using QUANTO program version 1.2.4, 1 May 2009 (https://keck.usc.edu/biostatistics/software/ accessed on 29 March 2026) software dedicated to epidemiology–genetics studies, under an unmatched case–control, gene-only, codominant model, assuming a minor-allele frequency of 0.3583, a population risk of acute rejection of 6.56% (35/533), and a two-sided α of 0.05. Student’s *t*-test was used to compare the mean age and nadir creatinine. For donor–recipient relationship, second- and third-degree relatives were combined for analysis, allowing for a more robust statistical evaluation. All results with a *p*-value that is less than 0.05 were considered statistically significant. Statistical analyses were performed using SPSS 31.0 software (IBM, Chicago, IL, USA).

## 5. Conclusions

This is the first genetic study investigating the genetic association of the *MIR146A* polymorphism rs2910164 and AR in a North African population. In this monocentric cohort of relatively young living-donor kidney transplant recipients with late AR, we did not detect a significant independent association between rs2910164 and AR after adjustment, including in sensitivity analyses using Firth penalized logistic regression. These findings suggest that rs2910164 is unlikely to exert a strong and consistent independent effect on AR risk in this specific setting. The fact that established clinical and immunological cofactors remained informative in our cohort, whereas rs2910164 did not show a significant independent association, further supports the interpretation that this polymorphism is unlikely to be a major determinant of AR in this context. However, more modest effects cannot be excluded, particularly given the specific clinical and phenotypic characteristics of this cohort. Our results therefore add to the interpretation of the conflicting literature by suggesting that any effect of this variant, if present, is likely to be modest, context-dependent, and influenced by population background and phenotype definition. Overall, rs2910164 does not appear to be a robust standalone biomarker of AR risk in this context. Larger, multicentric, and phenotypically well-stratified studies, particularly in North African populations, are needed to validate these findings and support future meta-analyses.

## Figures and Tables

**Table 1:** The demographic and clinical characteristics of the patients.

Demographic and Clinical Parameters	Acute Rejection (n = 35)	Non-Acute Rejection (n = 60)	*p*-Value	OR (95% CI)
**Donor and recipient age**				
Recipient age (mean, ±SD), years	27.51 ± 10.339	32.83 ± 9.758	0.014 *	
Donor age (mean, ±SD), years	49.57 ± 10.37	43.27 ± 10.266	0.005 *	
R-D age difference ± SD (years)	23.37 ± 11.29	14.83 ± 10.54	<0.001 *	
R-D age imbalance direction ^#^				
Donor age < recipient age	3 (8.6)	20 (33.3)	0.007 *	5.333 (1.454–19.559)
Donor age > recipient age	32 (91.4)	40 (66.7)		
**Donor and recipient sex**				
Recipient sex, No. (%)				
Male	21 (60%)	36 (60%)	0.98	1 (0.427–2.342)
Female	14 (40%)	24 (40%)		
Donor sex, No. (%)				
Male	13 (37%)	25 (42%)	0.664	0.864 (0.351–1.948)
Female	22 (63%)	35 (58%)		
**Recipient BMI**				
BMI, mean ± SD	21.69 ± 4.131	21.66 ± 3.38	0.971	
**Donor–recipient relationship (n, %)**				
Related (1st degree)	29 (82.9)	48 (80)	0.730	0.828 (0.280–2.444)
Related (2nd and 3rd degrees)	1 (2.9)	4 (6.7)	0.649	2.429 (0.261–22.639)
Unrelated	5 (14.3)	8 (13.3)	0.990	1.083 (0.325–3.612)
**HLA mismatch, No. (%)**				
HLA-A * (mismatch), No. (%)				
0 MM	4 (11.4)	5 (8.3)	0.721	0.705 (0.176–2.819)
1 MM	25 (71.4)	53 (88.3)	0.042 *	0.330 (0.113–0.969)
2 MM	6 (17.1)	2 (3.3)	0.048 *	6.000 (1.139–31.595)
Number of HLA mismatches				
<3 mismatches	25 (71.4)	50 (83.3)	0.175	2 (0.476–5.433)
≥3 mismatches	10 (28.6)	10 (16.7)		
**DSA ^##^, No. (%)**				
Positive	16 (45.7)	5 (8.3)	<0.001 *	9.263 (2.988–28.720)
Negative	19 (54.28)	55 (91.67)		
**Post-transplant viral infections, No. (%)**				
Presence of viral infection				
Infection	15 (42.85)	4 (6.67)	<0.001 *	10.5 (3.1–35.6)
No infection	20 (57.14)	56 (93.33)		

^#^ Age imbalance direction indicates whether the donor was younger than the recipient (donor age < recipient age) or older than the recipient (donor age > recipient age). D: donors, R: recipients, MM: mismatch, ^##^ DSA: preformed donor-specific antibody, * statistically significant (<0.05).

**Table 2:** Allele frequencies of rs2910164 (*MIR146A*) in AR and non-AR groups.

Group	G Allele n (%)	C Allele n (%)	HWE (χ^2^, *p*)
AR (n = 35)	49 (70.0%)	21 (30.0%)	0.46, *p* > 0.05
Non-AR (n = 60)	77 (64.1%)	43 (35.9%)	0.15, *p* > 0.05

**Table 3:** Distribution of rs2910164 genotype frequencies among AR group and non-AR group.

Genetic Model		Acute Rejection (n = 35), n (%)	Non-Acute Rejection (n = 60), n (%)	Unadjusted OR (95% CI)	*p*	Adjusted OR ^a^ (95% CI)	*p* ^a^	Firth OR ^b^ (95% CI)	*p* ^b^
Codominant	GG	18 (51.4)	24 (40)	1		1		1	
	GC	13 (37.1)	29 (48.3)	0.598 (0.244–1.463)	0.257	0.414 (0.119–1.444)	0.166	0.457 (0.133–1.45)	0.186
	CC	4 (11.4)	7 (11.7)	0.762 (0.193–3.005)	0.746	0.344 (0.055–2.149)	0.254	0.412 (0.068–2.123)	0.295
Dominant	GG	18 (51.4)	24 (40)						
	GC-CC	17 (48.6)	36 (60)	0.630 (0.272–1.459)	0.280	0.396 (0.123–1.273)	0.120	0.436 (0.136–1.293)	0.135
Recessive	GG-GC	31 (88.6)	63 (88.3)						
	CC	4 (11.4)	7 (11.7)	0.977 (0.265–3.606)	0.990	0.525 (0.095–2.901)	0.460	0.601 (0.113–2.812)	0.525
Overdominant	GG-CC	22 (62.9)	31 (51.7)						
	GC	13 (37.1)	29 (48.3)	0.632 (0.269–1.481)	0.288	0.526 (0.163–1.704)	0.284	0.564 (0.176–1.683)	0.307

OR ^a^: adjusted by standard logistic regression for donor–recipient age difference, post-transplant viral infections, mismatches, and preformed donor-specific antibodies. OR ^b^: adjusted by Firth penalized logistic regression using the same covariates as a sensitivity analysis.

**Table 4:** The demographic and clinical characteristics of the patients after stratification of the AR group.

Demographic and Clinical Parameters	ABMR AR (n = 25) (A)	TCMR AR (n = 10) (B)	Non-AR (n = 60) (C)	*p*-Value, *OR (95% CI)* (A) vs. (C)	*p*-Value, *OR (95% CI)* (B) vs. (C)
**Donor and recipient age**					
Recipient age (mean, ±SD), years	26.92 ± 10.78	29 ± 9.51	32.83 ± 9.758	0.016 *	0.253
Donor age (mean, ±SD), years	50.28 ± 10.26	47.8 ± 10.96	43.27 ± 10.266	0.005 *	0.205
R-D age difference ± SD (years)	24.32 ± 11.739	21 ± 10.28	14.83 ± 10.54	<0.001 *	0.09
R-D age imbalance direction ^#^					
Donor age < recipient age	2 (8%)	1 (10%)	20 (33.3%)	0.009 * 5.75 (1.231–26.858)	0.262 4.5 (0.532–38.04)
Donor age > recipient age	23 (92%)	9 (90%)	40 (66.7%)		
**DSA ^##^, No. (%)**					
Positive	10 (40%)	6 (60%)	5 (8.3%)	0.001 * 7.333 (2.174–24.737)	0.001 * 16.5 (3.462–78.65)
Negative	15 (60%)	4 (40%)	55 (91.67%)		
**Post-transplant viral infections, No. (%)**					
Presence of viral infection (%)					
Infection	12 (48%)	3 (30%)	4 (6.67%)	*p* < 0.001 * 12.923 (3.584–46.592)	0.055 6 (1.106–32.537)
No infection	13(52%)	7(70%)	56 (93.33%)		

ABMR: antibody-mediated rejection, TCMR: T-cell-mediated rejection. ^#^ Age imbalance direction indicates whether the donor was younger than the recipient (donor age < recipient age) or older than the recipient (donor age > recipient age). D: donors, R: recipients, ^##^ DSA: preformed donor-specific antibody, * statistically significant (<0.05). Only characteristics with a significant difference are shown.

## Data Availability

The new data underlying this article are available in the article.

## Supplementary Materials

The following supporting information can be downloaded at https://www.mdpi.com/article/10.3390/ijms27115105/s1.

## Abbreviations

AA	African American
ABMR	Antibody-mediated rejection
Ago	Argonaute proteins
ALT	Alternate
AR	Acute rejection
BK virus	Human polyomavirus 1
BMI	Body Mass Index
CI	Confidence interval
CMV	Cytomegalovirus
DNA	Deoxyribonucleic acid
DRAG	Donor–recipient age gradient
DSAs	Donor-specific antibodies
EA	European American
EDTA	Ethylenediaminetetraacetic acid
HLA	Human Leukocyte Antigen
HWE	Hardy–Weinberg equilibrium
IFN-γ	Interferon-gamma
IRAK1	Interleukin-1 Receptor-Associated Kinase1
KTR	Kidney transplant recipient
LD	Linkage disequilibrium
MAF	Minor-allele frequency
MM	Mismatch
NF-κB	Nuclear factor kappa B
OR	Odds Ratio
PCR	Polymerase chain reaction
R-D	Recipients–donors
REF	Reference
RISC	RNA-induced silencing complex
RNA	Ribonucleic acid
RNase III	Ribonuclease III
SARS-CoV-2	Severe acute respiratory syndrome coronavirus 2
SNP	Single-nucleotide polymorphism
SPSS	Statistical Package for the Social Sciences
TCMR	T-cell-mediated rejection
TLR	Toll-like receptor
TNF-α	Tumor Necrosis Factor-alpha
TRAF6	TNF Receptor-Associated factor 6
UTR	Untranslated Region
