# Supplementary material for: Population-Specific Exploration of MIR146A Gene Polymorphism in Acute Renal Rejection: A Cross-Sectional, Case–Control Study
Source: Int J Mol Sci. 2026 Jun 4;27(11):5105. doi: 10.3390/ijms27115105 (PMC13257254; doi:10.3390/ijms27115105)
Supplement: Supplementary file 1 [file ijms-27-05105-s001.zip › ijms-4259526-supplementary.pdf]

Supplementary Material

Table S1. The demographic and clinical characteristics of the patients.

Demographic and clinical parameters	Acute rejection (n = 35)	Non-acute rejection (n = 60)	*p* value	*OR (95% CI)*
**Donors and Recipients sex**				
Donor-Recipient sex match, No. (%)				
Same-sex transplantation	15(42.9)	29 (48.3)		
Cross-sex transplantation	20(57.1)	31 (51.7)	0.267	1.247 (0.539-2.887)
Donor-Recipient sex combinations, No. (%)				
Male → Female	6(17.15)	10(16.7)	0.952	1.034(0.341-3.141)
Male → Male	7(20)	15(25)	0.574	0.75(0.272-2.067)
Female → Male	14(40)	21(35)	0.627	1.238(0.524-2.924)
Female → Female	8(22.85)	14(23. 3)	0.958	0.974(0.362-2.62)
**Recipients pretransplant medical disorders and interventions**				
Blood hypertension	11(31.4)	17(28.3)	0.750	1.159 (0.468-2.875)
Diabetes	1(2.9)	1(1.7)	0.990	1.735 (0.105-28.643)
Autoimmune diseases	2(5.7)	2(3.3)	0.624	1.758 (0.236-13.065)
Bacterial infections	4(11.4)	4(6.7)	0.461	1.806 (0.422-7.730)
Blood diseases (Beta thalassemia)	1(2.9)	0	0.368	1.029 (0.973-1.090)
No medical disorders	16(45.7)	36(60)	0.177	1.781 (0.767-4.134)
Pre-transplant blood transfusions	6(17.1)	10(16.7)	0.952	1.034 (0.341-3.141)
Dialysis duration before transplantation (years), mean ± SD	3.57±2.07	2.73±3.08	0.180	
**HLA mismatch, No. (%)**				
HLA/B* (mismatch) No. (%)				
0 MM	2(5.7)	3(5)	0.990	0.868 (0.138-5.467)
1 MM	26(74.3)	47(78.3)	0.653	0.799 (0.301-2.120)
2 MM	7(20)	10(16.7)	0.684	1.250 (0.428-3.648)
HLA/DRB1* (mismatch) No. (%)				
0 MM	5(14.3)	9(15)	0.924	1.059 (0.325-3.455)
1 MM	26(74.3)	45(75)	0.938	0.963 (0.370-2.508)
2MM	4(11.4)	6(10)	0.990	1.161 (0.304-4.435)
**Post-transplant viral infections, No (%)**				
Type of viral infection ##				
No Infection	20 (57.14)	56 (93.33)		
BK Virus	2(5.7)	3(5)	0.990	1.87 (0.28 - 12.6)
CMV infection	5(14.3)	0 (0)	0.003*	30.3 (1.7 - 540)
SARS-COV2 infection	8(22.9)	1(1.7)	0.002*	22.4 (2.6 - 193.3)
**Renal function parameters, Mean±SEM (µmol/L)**				
Nadir creatinine	114.0 ± 3.5	97.2 ± 1.8	0.000*	-
Creatinine at rejection episode	574.6 ± 46.9	N/A		

MM: mismatch, R: Recipients, D: Donors, BK virus: Human polyomavirus 1, CMV: cytomegalovirus
* Statistically significant (<0.05), SEM: Standard Error of the Mean, N/A: Non-Applicable, ## Comparison was done with cases without infection.
